# Hybrid Pharmacophore- and Structure-Based Virtual Screening Pipeline to Identify Novel EGFR Inhibitors That Suppress Non-Small Cell Lung Cancer Cell Growth

**DOI:** 10.3390/ijms23073487

**Published:** 2022-03-23

**Authors:** Chia-Wei Weng, Chi-Hsuan Wei, Jeng-Yuan Tsai, Yi-Hua Lai, Gee-Chen Chang, Jeremy J. W. Chen

**Affiliations:** 1 Institute of Biomedical Sciences, National Chung Hsing University, Taichung 40227, Taiwan; cwweng060304@gmail.com (C.-W.W.); crystalwei823@gmail.com (C.-H.W.); imjie6@msn.com (J.-Y.T.); 2 Institute of Medicine, Chung Shan Medical University, Taichung 40201, Taiwan; 3 Rheumatology and Immunology Center, China Medical University Hospital, Taichung 40447, Taiwan; beaty081533@gmail.com; 4 College of Medicine, China Medical University, Taichung 40402, Taiwan; 5 Rheumatic Diseases Research Center, China Medical University Hospital, Taichung 40447, Taiwan; 6 Division of Pulmonary Medicine, Department of Internal Medicine, Chung Shan Medical University Hospital, Taichung 40201, Taiwan; 7 School of Medicine, Chung Shan Medical University, Taichung 40201, Taiwan; 8 Biotechnology Center, National Chung Hsing University, Taichung 40227, Taiwan; 9 Institute of Molecular Biology, National Chung Hsing University, Taichung 40227, Taiwan

**Keywords:** lung cancer, EGFR inhibitor, pharmacophore model, virtual screening, molecular docking

## Abstract

Dysregulated epidermal growth factor receptor (EGFR) expression is frequently observed in non-small cell lung cancer (NSCLC) growth and metastasis. Despite recent successes in the development of tyrosine kinase inhibitors (TKIs), inevitable resistance to TKIs has led to urgent calls for novel EGFR inhibitors. Herein, we report a rational workflow used to identify novel EGFR-TKIs by combining hybrid ligand- and structure-based pharmacophore models. Three types of models were developed in this workflow, including 3D QSAR-, common feature-, and structure-based EGFR-TK domain-containing pharmacophores. A National Cancer Institute (NCI) compound dataset was adopted for multiple-stage pharmacophore-based virtual screening (PBVS) of various pharmacophore models. The six top-scoring compounds were identified through the PBVS pipeline coupled with molecular docking. Among these compounds, NSC609077 exerted a significant inhibitory effect on EGFR activity in gefitinib-resistant H1975 cells, as determined by an enzyme-linked immunosorbent assay (ELISA). Further investigations showed that NSC609077 inhibited the anchorage-dependent growth and migration of lung cancer cells. Furthermore, NSC609077 exerted a suppressive effect on the EGFR/PI3K/AKT pathway in H1975 cells. In conclusion, these findings suggest that hybrid virtual screening may accelerate the development of targeted drugs for lung cancer treatment.

## 1. Introduction

Receptor tyrosine kinases (RTKs) play pivotal roles in the regulation of developmentally relevant signal transduction events [1,2]. Abnormal RTK activity is one of the leading factors correlated with the carcinogenesis of different cancer types and therefore, has become an attractive therapeutic focus of targeted cancer therapy [3,4]. The epidermal growth factor receptor (EGFR) family is one of the most studied TK targets because it plays vital roles in mediating cell growth, differentiation, and survival signaling [5,6]. The EGFR family consists of four distinct receptors: EGFR (also known as HER1 or ErbB1), HER2 (ErbB2), HER3 (ErbB3), and HER4 (ErbB4) [5,7,8]. EGFR is a 170-kDa plasma membrane glycoprotein with a cytoplasmic TK domain, a single hydrophobic transmembrane segment, and an extracellular ligand-binding domain, which is similar to other receptors in the ErbB family [5,8]. Several ligands, such as EGF and transforming growth factor (TGF), regulate ligand interactions with the extracellular domain of EGFR, leading to receptor dimerization and stabilization. The TK domain in EGFR catalyzes the transfer of a γ-phosphate bound to ATP to tyrosine residues at intracellular autophosphorylation sites in the carboxy-terminal tail, which ultimately leads to the activation of numerous downstream signaling pathways [9,10].

Aberrant EGFR activation frequently occurs in diverse malignancies, including lung cancer [11,12,13], and triggers a series of tumorigenic events, such as cell proliferation, cell invasion, and metastasis and angiogenesis [14,15,16]. Lung cancer is the leading cause of cancer-related mortality worldwide [17]. Non-small cell lung cancer (NSCLC) accounts for approximately 80% of lung cancers, and it has an overall 5-year survival rate of 15% [18]. One of the clinical strategies for targeting EGFR activation in advanced NSCLC is small-molecule inhibition of the catalytic kinase domain. To date, two 4-anilinoquinazoline derivatives (gefitinib [19] and erlotinib [20]), which are reversible ATP competitive inhibitors that prevent the autophosphorylation of EGFR TK, have been clinically approved. In addition, a 4-anilinoquinazoline derivative (afatinib [21,22]) and a mono-anilino-pyrimidine compound (osimertinib [23]) lead to noncompetitive and irreversible inhibition via covalent bond formation with the ATP-binding site in the EGFR-TK domain.

Previous studies have shown that the first-generation EGFR-TKIs gefitinib and erlotinib lead to better outcomes in approximately 5–15% of NSCLC patients with sensitizing EGFR mutations (exon 19 deletions and exon 21 L858R substitution) than in those with wild-type EGFR [24,25,26]. However, approximately 50% of patients have been found to have EGFR exon 20 T790M mutations at the time of acquired resistance to first-generation TKI therapy [27]. Second- and third-generation TKIs (i.e., afatinib and osimertinib) are currently approved for use in metastatic EGFR T790M mutant-positive NSCLC [28,29]; however, these two medicines unavoidably cause severe on-target side effects [30] and acquired resistance mutations in EGFR exon 20 C797S [31] and G796D [32]. Resistance to EGFR-TKIs is a major hindrance to NSCLC treatment success; therefore, novel therapeutic agent development is an urgent goal.

Given the reversible and irreversible molecules targeting the EGFR-TK domain, developing predictive models describing the crucial chemical features of these molecules is clearly needed. In this study, qualitative and quantitative pharmacophore models were developed based on the pharmacophore features of EGFR inhibitors. Structure-based pharmacophore features were also determined on the basis of the drug-binding pocket in the mutant EGFR-TK domain. Subsequently, sequential screening was performed to identify the novel EGFR inhibitor candidates through pharmacophore models and biological assays.

## 2. Results

### 2.1. Workflow

**Figure 1:**
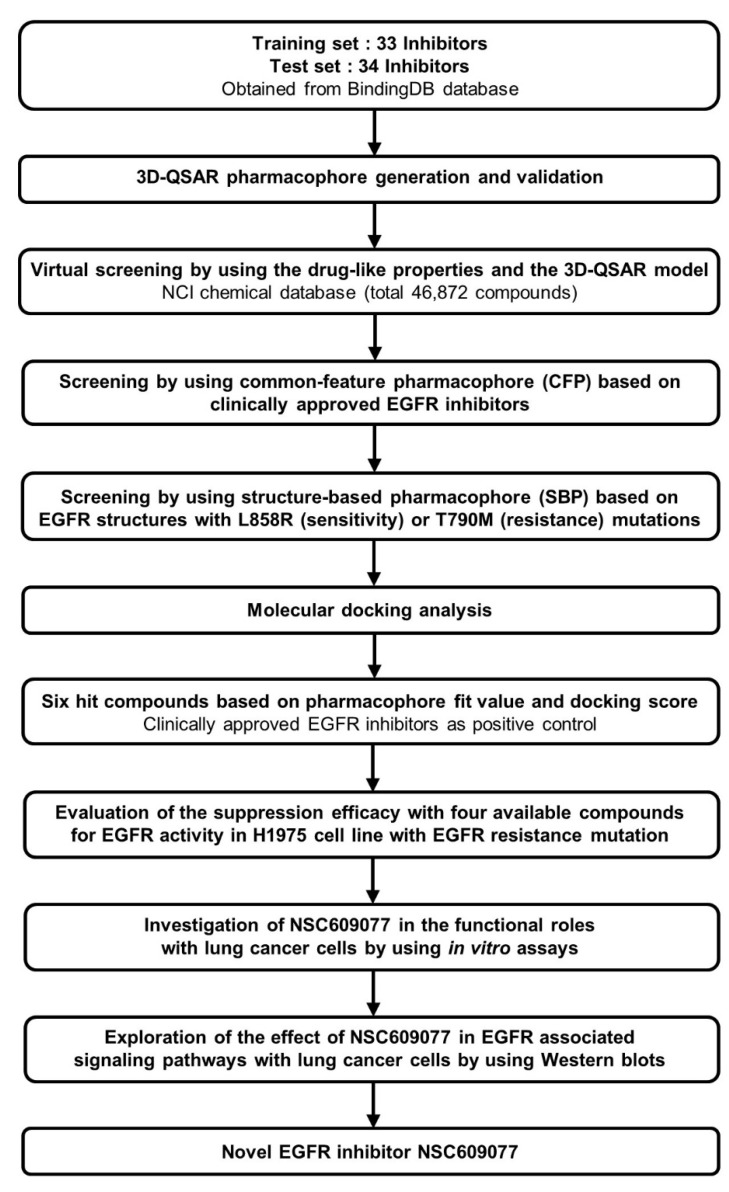
The overall scheme of multiple-stage pharmacophore-based virtual screening (PBVS) implemented for the identification of the novel EGFR inhibitor NSC609077. Abbreviations- 3D-QSAR: 3D quantitative structure-activity relationship, NCI: National Cancer Institute, *EGFR*: Epidermal growth factor receptor.

Accelrys Discovery Studio 3.5 (DS) (Accelrys Inc., San Diego, CA, USA) software was used to perform all the calculations in this study. A CHARMM force field [33] was utilized for energy minimization during the calculation processes. A set of 67 chemical compounds and their half maximum inhibitory concentration (IC_50_) values determined through a kinase inhibition assay were obtained from the BindingDB database [34] and rationally categorized into training and test sets of 33 and 34 compounds, respectively. The 3D quantitative structure-activity relationship (QSAR) pharmacophore was modeled using these compounds and was prioritized as the 3D structure query in the National Cancer Institute (NCI) chemical database [35]. Subsequently, one common feature pharmacophore (CFP) from among the clinical EGFR inhibitors, and two structure-based pharmacophores (SBPs) dependent on mutant EGFR structures were constructed through qualitative methods and adopted in further multiple-stage pharmacophore-based virtual screenings (PBVS). Furthermore, molecular docking was performed to refine the retrieved hits. The six top-scoring hits were chosen as candidate compounds. Biological validation with ELISA was then performed to evaluate the efficacy of EGFR activity suppression of the final hits identified through this pipeline. Finally, we determined that NSC609077 is a candidate inhibitor that exerted a significant effect on EGFR inhibition. Moreover, we investigated the functional roles and associated signaling pathways underlying the ability of NSC609077 to suppress lung cancer progression in vitro. The complete workflow is summarized in Figure 1.

### 2.2. 3D QSAR Pharmacophore Generation and Validation

**Figure 2:**
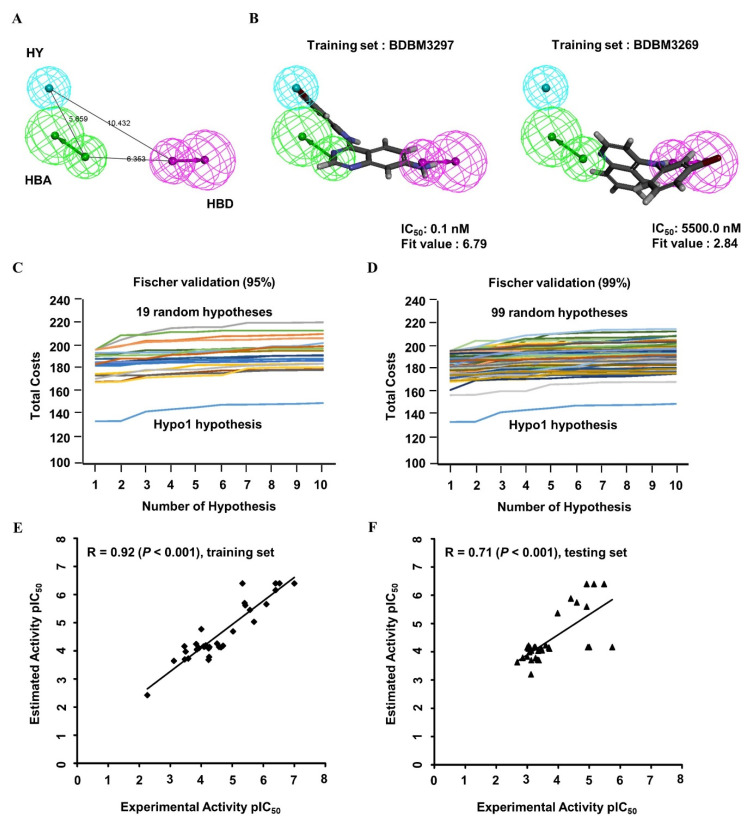
The 3D QSAR Hypo1 pharmacophore model of EGFR inhibitors. (**A**) Pharmacophore features of the Hypo1 model: hydrogen bond acceptor (HBA, green), hydrogen bond donor (HBD, purple), and hydrophobic region (HY, blue). The distance (Å) between every two features is shown. (**B**) The most active compound BDBM3297 (half maximum inhibitory concentration, IC_50_ = 0.1 nM; fit value = 6.79) and the most inactive compound BDBM3269 (IC_50_ = 5500 nM; fit value = 2.84) were mapped to the Hypo1 model. BDBM number: the general identifier of compound in the BindingDB database. (**C**,**D**) Validation of the statistical significance of the 3D QSAR Hypo1 model. Fischer’s randomization test was used to examine significant differences in total costs between the Hypo1 model and random pharmacophore models at a 95% confidence level (**C**) and 99% confidence level (**D**). (**E**,**F**) Evaluation of the predictive efficacy of the 3D QSAR Hypo1 model. Correlation plot between the experimental activity and the predicted activity of the 3D QSAR training set compounds (E; r = 0.92, *p* < 0.001) and test set compounds (F; r = 0.71, *p* < 0.001) when mapped to the Hypo1 model. The pIC_50_ values of training set compounds and test set compounds were shown with the black square and the black triangle, respectively. pIC_50_: the negative logarithm value of the IC_50_ value when converted to millimolar.

**Figure A1:**
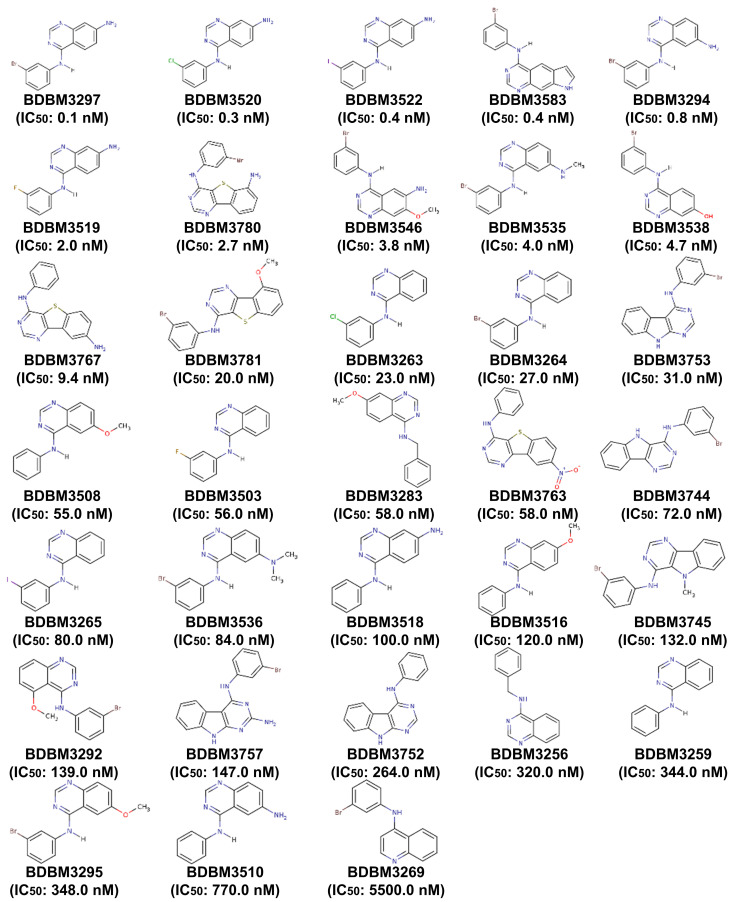
The chemical structures of 33 EGFR inhibitors comprising the training set for the construction of the 3D QSAR pharmacophore model.

The 3D QSAR pharmacophore model was constructed on the basis of 33 structurally diverse training set compounds with IC_50_ values spanning five orders of magnitude (Appendix A; Figure A1), which constitute a requirement for large-scale virtual screening. The dataset activity (based on the IC_50_) was roughly classified into three groups: highly active (IC_50_ < 100 nM), moderately active (100 nM ≤ IC_50_ < 1000 nM), and nominally active (IC_50_ ≥ 1000 nM). Four types of crucial features, namely, hydrogen bond acceptors (HBAs), hydrogen bond donors (HBDs), hydrophobic regions (HYs), and aromatic rings (ARs), were investigated among the training set compounds, and the hypothetical pharmacophore was modeled. The 10 highest-ranked hypothetical pharmacophores with the corresponding statistical parameters were constructed to illustrate different constituents contributing to the pharmacophoric features and to reflect the significance of each model; these results are shown in Table 1. The top-ranked hypothetical structure (i.e., Hypo1) was chosen as the best model because of its parameters: the highest correlation coefficient (0.92), the lowest root mean square deviation (RMS; 0.88), the lowest total cost (136.11), the most reliable configuration cost (10.43), and the highest cost difference (60.98). Moreover, the null cost and the fixed cost were 197.09 and 122.56, respectively. Hypo1 was composed of HBAs, HBDs, and HYs, as shown in Figure 2A. The distance between every two chemical features ranged from approximately 5.7 to 10.4 Å. The most active compound, BDBM3297 (IC_50_, 0.1 nM), showed a higher fit value of 6.79 when mapped to Hypo1, while the least active compound, BDBM3269 (IC_50_, 5500 nM), displayed a low fit value, 2.84, as shown in Figure 2B.

To validate the statistical significance of the Hyop1 model, Fischer’s randomization test was performed through the CatScramble method to shuffle the IC_50_ values of the training set compounds and construct a randomly generated hypothetical pharmacophore. A total of 19 and 99 random hypothetical models were generated at the 95 and 99% confidence levels, respectively. The total costs of the randomly generated hypothetical pharmacophores were much higher than those of the original Hypo1 hypothetical model at these two confidence levels (Figure 2C,D). The results of the randomization test indicated that Hypo1 was not generated on the basis of the random correlations.

**Figure A2:**
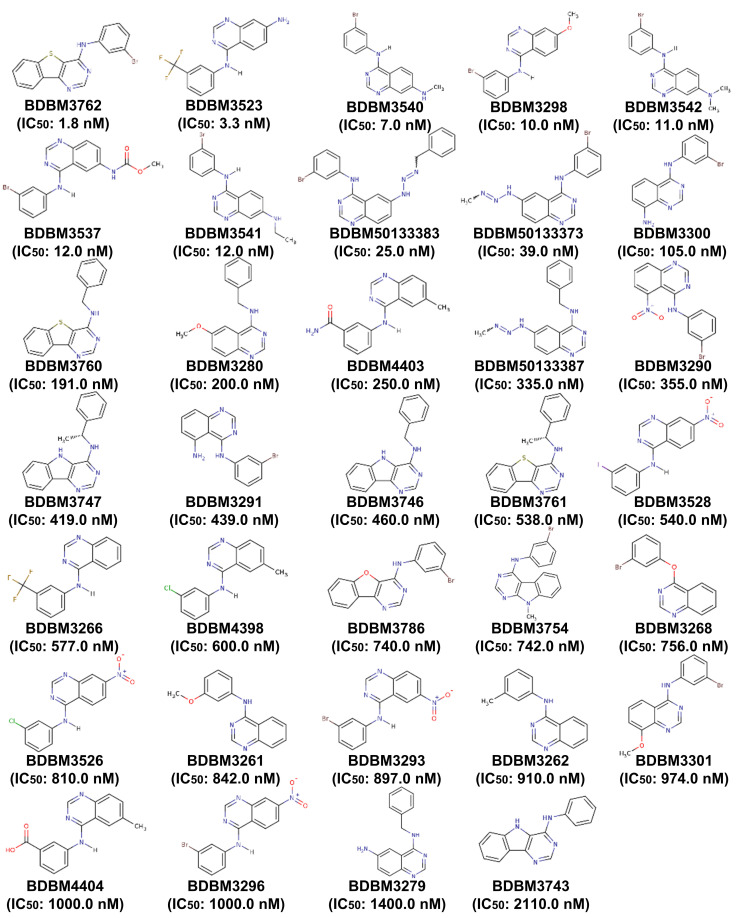
The structures of 34 structurally diverse compounds comprising the test set used to evaluate the validity of the 3D QSAR pharmacophore model.

To validate the predictive efficacy of the Hypo1 model, the correlation between the experimental and estimated IC_50_ values of the training set molecules indicated good predictability, as shown in Figure 2E (r = 0.92, *p* < 0.001). Furthermore, 20 of 22 highly active, 4 of 10 moderately active, and 1 nominally active training set compounds were estimated in the same order of magnitude (Table 2). In addition, the 34 tested set compounds with IC_50_ values spanning five orders of magnitude were tested for their predictability potential (Appendix A; Figure A2). The test set evaluation with the hypothetical Hypo1 model showed low but significant predictive ability with a correlation coefficient value of 0.71 (*p* < 0.001) (Figure 2F). Collectively, these data showed a suitable correlation between the experimental and estimated activities of the Hypo1 training and test set molecules. All the validation approaches demonstrated that the Hypo1 model has appropriate discriminatory power and can be adopted as the best 3D QSAR model for virtual screening.

### 2.3. Identification of Common Feature Pharmacophores

**Figure 3:**
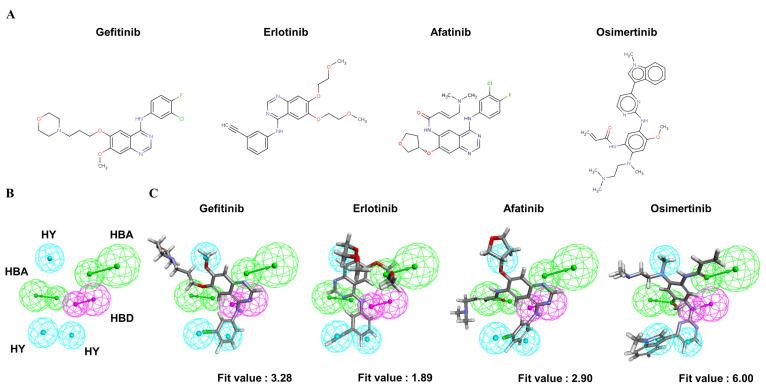
The common feature pharmacophore model of clinical EGFR inhibitors. (**A**) Structures of four clinically approved EGFR inhibitors. Gefitinib, erlotinib, and afatinib are 4-anilinoquinazoline derivatives. Osimertinib is a mono-anilino-pyrimidine derivative. (**B**) Pharmacophore features of the Pharm2 model: hydrogen bond acceptor (HBA, green), hydrogen bond donor (HBD, purple), and hydrophobic region (HY, blue). (**C**) Mapping of gefitinib (fit value = 3.28), erlotinib (fit value = 1.89), afatinib (fit value = 2.90), and osimertinib (fit value = 6.00) in the Pharm2 model.

To understand the crucial pharmacophore features of the reversible and irreversible EGFR inhibitors, four clinically approved small-molecule drugs (Figure 3A) were used to identify the common chemical features among them. Through the modeling processes, the 10 highest-ranked hypothetical pharmacophores were produced, as shown in Table 3. To identify the optimal model from among the ten hypothetical models, the following four parameters were the criteria: (1) a high rank value indicated greater validity of the produced model; (2) a perfect direct hit indicated complete matching of the produced model to a training set compound; (3) a partial hit indicated incomplete matching of the model to a training molecule(s); and (4) a max hit value represented the number of mapped chemical features. According to these criteria, the Pharm2 model, shown in Table 3, was chosen as the optimal model on the basis of the following parameters: (i) the second-highest rank value of 53.04; (ii) a perfect hit of the four tested EGFR inhibitors; (iii) no partial hits among the four tested inhibitors; and (iv) the highest max hit value. The Pharm2 hypothetical model was composed of three HYs, one HBD, and two HBAs, as shown in Figure 3B. As illustrated in Figure 3C, the four EGFR inhibitors were mapped to the Pharm2 model with the following derived pharmacophoric fit values: gefitinib (fit value, 3.28), erlotinib (fit value, 1.89), afatinib (fit value, 2.90), and osimertinib (fit value, 6.00).

### 2.4. Generation of Structure-Based Pharmacophores

**Figure 4:**
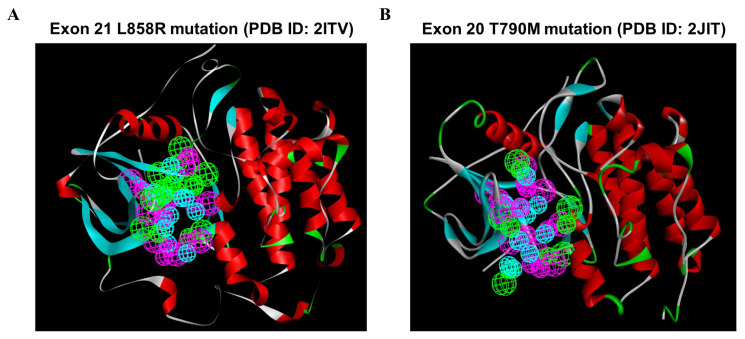
The structure-based pharmacophoric features involving the ATP-bound pocket of the EGFR L858R and T790M structures. Pharmacophore features were identified on the basis of the ATP-binding site in the EGFR kinase domain: hydrogen bond acceptor (HBA, green), hydrogen bond donor (HBD, purple), and hydrophobic region (HY, blue). (**A**) A total of 8 HBAs, 8 HBDs, and 12 HYs were identified based on the crystal structure of the EGFR L858R mutant. (**B**) A total of 5, 8, and 10 features were identified based on the crystal structure of the EGFR T790M mutant and classified into three types of features: HBAs, HBDs, and HYs. Abbreviations- PDB: Protein Data Bank.

To investigate the most comprehensive pharmacophoric features, EGFR L858R and T790M mutants were adopted, and the key chemical features were discerned by examining the residues in the ATP-competitive pocket of the kinase domain. A total of 246 HBAs, 230 HBDs, and 88 HYs were identified in the EGFR L858R mutant. After a cluster analysis of these features was performed, the optimal SBP was generated; the set of features included 8 HBAs, 8 HBDs, and 12 HYs (Figure 4A). In addition, a total of 73 HBAs, 264 HBDs, and 102 HYs were identified in the EGFR T790M mutant. Through clustering, the crucial features of the 5 HBAs, 8 HBDs, and 10 HYs were found to be retained in the optimal SBP model corresponding to the T790M mutant (Figure 4B). Subsequently, these two SBP models, the Hypo1 3D QSAR model and the Pharm2 CFP model, were utilized for multiple-stage PBVS.

### 2.5. Identification of Candidate Compounds through Multiple-Stage PBVS

To identify potential EGFR inhibitors, 46,872 NCI compounds were screened by multiple-stage PBVS coupled with molecular docking. Initially, drug-like properties characterized through criteria such as Lipinski’s rule of five [36] and Veber’s rules [37] were preferentially assessed to explore the druggability of the candidate molecules. By discerning the druggable properties in this manner, 38,546 compounds with good drug likeness were chosen. To identify the most potent inhibitors against the EGFR-TK domain, first-, second-, and third-generation EGFR inhibitors were adopted as positive controls in the calculation of the pharmacophore fit values corresponding to three types of pharmacophore models; the results are shown in Table A1 (Appendix A). Among the four clinically approved medicines, erlotinib presented the lowest degree of overlap with each pharmacophore model. To prevent the loss of additional candidates, we selected three types of fit values derived from the erlotinib molecule as criteria for screening. Then, the selected drug-like compounds were mapped to the Hypo1 model, which was based on the 3D QSAR pharmacophore, to calculate the fit values, and 16,666 hit compounds had fit values larger than the control value (the fit value for the 3D QSAR pharmacophore model was 3.17). Then, the 16,666 hit compounds were subsequently mapped to the Pharm2 model on the basis of the CFP to calculate fit values, and 2682 hits were identified based on the fit values derived by mapping erlotinib to the Pharm2 CFP model (the fit value was 1.89). The retained hit compounds were mapped to the two individual SBP models, and the fit values of eight hit compounds exceeded the control values (the fit value of the SBP derived from the EGFR L858R mutant was 1.50, and the fit value of the SBP derived from the EGFR T790M mutant was 1.15).

Although the retained hit compounds were considered to represent the crucial pharmacophore features derived from the EGFR L858R and T790M mutant structures, molecular docking analysis was still performed to understand the binding modes and select six models that had a LibDock score higher than the control score (the LibDock score for the EGFR L858R mutant was 111.59, and the LibDock score of the EGFR T790M mutant was 106.28, respectively). Among the six hit compounds, four compounds obtained through the NCI open chemicals repository were employed to demonstrate the validity of multiple-stage PBVS. Therefore, their inhibitory effects on EGFR activity were evaluated by performing an ELISA to measure the phosphorylation level of the autophosphorylation site (i.e., the EGFR Y1068 residue) in gefitinib-resistant H1975 lung cancer cells. The ELISA results showed that at a 1 µM concentration, each compound had a maximum inhibitory rate on EGFR activity greater than 50%, except for NSC7521. Among these compounds, NSC609077 showed the most significant inhibition (an inhibitory rate of 79%) compared to the vehicle control. The detailed pharmacophore fit values, LibDock scores, and inhibitory rate on EGFR activity of the candidate compounds are shown in Table 4.

### 2.6. NSC609077 Inhibits the Anchorage-Dependent Growth and Motility of Lung Cancer Cells

**Figure 5:**
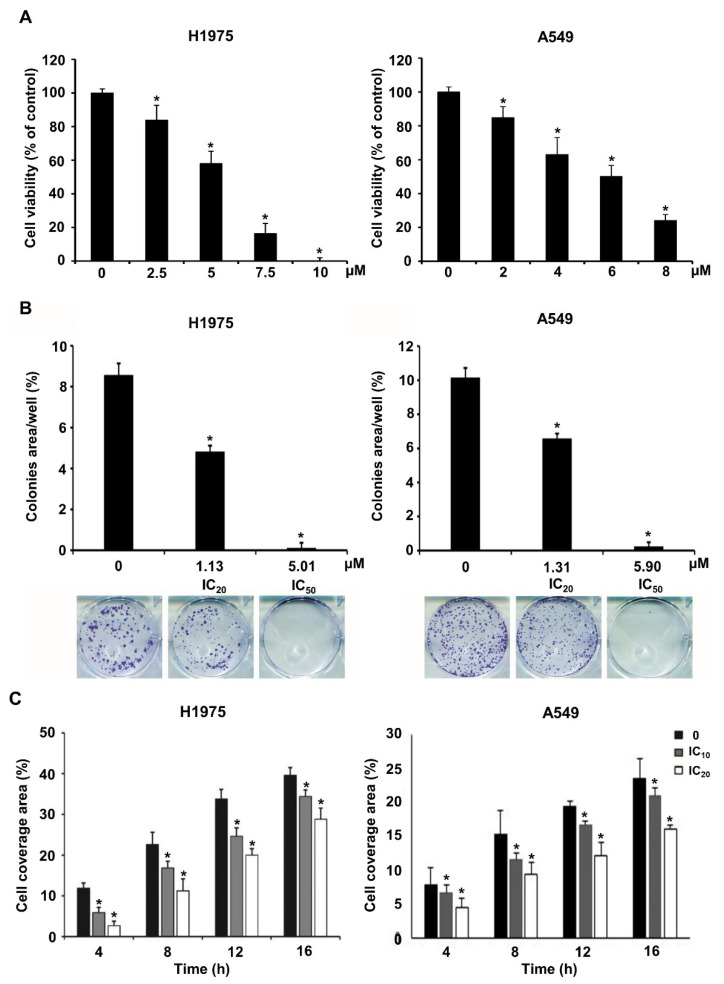
The impact of NSC609077 on lung cancer cell viability, clonogenicity, and motility. (**A**) The cytotoxicity of NSC609077 was evaluated in H1975 and A549 lung cancer cells with a cell viability assay. The results are shown as the percentages with respect to the control response (0 µM). (**B**) H1975 and A549 cells grown under anchorage-dependent conditions were treated with IC_20_ and IC_50_ of NSC609077 for 48 h and then evaluated through clonogenic assays. Quantification of the percentage of the colony-covered area of each treatment group, as assessed with ImageJ software. Representative images are shown. (**C**) The effect of IC_10_ (H1975, 0.47 µM; A549, 0.55 µM) and IC_20_ (H1975, 1.13 µM; A549, 1.31 µM) of NSC609077 treatment for 16 h on H1975 and A549 cell migration was assessed by scratch-wound assay. The percentage of the cell coverage areas was measured. * *p* < 0.05 compared with the vehicle-treated control.

As a demonstration of the validity of multiple-stage PBVS, NSC609077 was chosen to examine its efficacy as a functional antitumor drug. To determine the IC_50_ of NSC609077 before performing other experiments, we conducted a cytotoxicity assay after treating H1975 and A549 lung cancer cells with various concentrations of NSC609077 for 48 h. The analysis showed that the survival rates of both cell lines were significantly decreased in an appropriate dose-dependent manner (Figure 5A). The IC_20_ and IC_50_ values of NSC609077 in H1975 cells were calculated with CalcuSyn software to be 1.13 µM and 5.01 µM after 48 h of treatment, respectively. In addition, the IC_20_ and IC_50_ values of NSC609077 in A549 cells were 1.31 and 5.9 µM. Our results indicated that NSC609077 exhibited a better inhibitory effect on gefitinib-resistant H1975 cells harboring the EGFR T790M mutation than on A549 cells harboring wild-type EGFR. To investigate the antitumor effect of NSC609077, colony formation assays and scratch-wound assays were carried out in this study. The colony formation ability of both lung cancer cell lines was significantly inhibited by 48-h treatment with IC_20_ and IC_50_ concentrations of NSC609077 in a dose-dependent manner (Figure 5B). Because the formation of most of colonies in both cell lines was inhibited at the IC_50_ dosage of NSC609077, to enable the observation of the motility of both cell types, for the wound healing experiment, lower doses (IC_10_ and IC_20_) of NSC609077 were used at the indicated time points (4–16 h). Herein, NSC609077 significantly inhibited the migration of both lung cancer cell lines, especially at the IC_20_ does (Figure 5C). Taken together, our results showed that NSC609077 significantly inhibited the growth and migration of gefitinib-resistant or gefitinib-insensitive lung cancer cells.

### 2.7. Effects of NSC609077 on an EGFR-Associated Signaling Pathway

**Figure 6:**
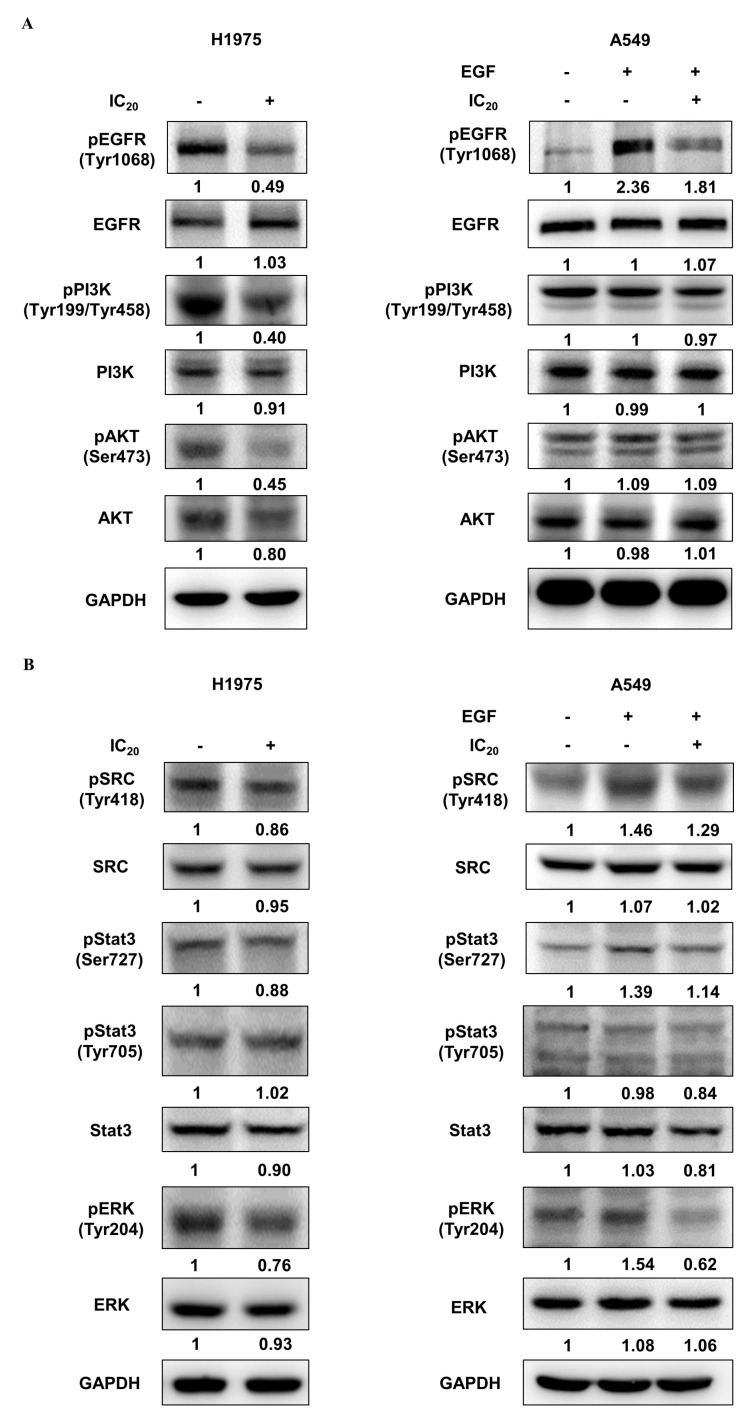
The effect of NSC609077 on an EGFR-associated signaling pathway in H1975 and A549 lung cancer cells. (**A**) Western blot showing EGFR, PI3K, and AKT in H1975 and A549 cells treated with the IC_20_ (H1975, 1.13 µM; A549, 1.31 µM) of NSC609077 for 48 h. (**B**) Western blot showing Src, STAT3, and ERK in H1975 and A549 cells treated with the IC_20_ of NSC609077 for 48 h. A549 cells with or without EGF stimulation. GAPDH served as the loading control. Protein expression was quantified with ImageJ software, and these data are shown below the gel graph. Abbreviations of genes/aliases- *EGFR*: Epidermal growth factor receptor, pEGFR: Phosphorylation of epidermal growth factor receptor, *PI3K*: Phosphatidylinositol-4,5-bisphosphate 3-kinase catalytic subunit beta, pPI3K: Phosphorylation of phosphatidylinositol-4,5-bisphosphate 3-kinase catalytic subunit beta, *AKT*: AKT serine/threonine kinase 1, pAKT: Phosphorylation of AKT serine/threonine kinase 1, *SRC*: SRC proto-oncogene, pSRC: Phosphorylation of SRC proto-oncogene, *Stat3*: Signal transducer and activator of transcription 3, pStat3: Phosphorylation of signal transducer and activator of transcription 3, *ERK*: Mitogen-activated protein kinase 1, pERK: Phosphorylation of mitogen-activated protein kinase 1, *GAPDH*: Glyceraldehyde-3-phosphate dehydrogenase.

Previous studies have reported that the resistance of first-generation EGFR-TKIs in NSCLC cells can be overcome by suppressing the EGFR/PI3K/AKT signaling pathway [38,39,40]. To further confirm the inhibitory effect of NSC609077 on the expression of the EGFR/PI3K/AKT axis, H1975 and A549 lung cancer cells were treated with the respective IC_20_ dose of NSC609077 for 48 h; Western blot analysis was then performed. As shown in Figure 6A, significant inhibitory effects of the IC_20_ dose of NSC609077 on the phosphorylation of EGFR, PI3K, and AKT were detected in the H1975 cells. Furthermore, the total protein expression levels of the axis components, except for the EGFR level, were slightly reduced. In contrast, no significant inhibition of the levels of total or phosphorylated proteins in this axis was found in NSC609077-treated A549 cells exposed to EGF stimulation; however, EGFR phosphorylation was reduced. Given this evidence, NSC609077 may not inhibit the growth or migration of gefitinib-insensitive A549 lung cancer cells by attenuating EGFR/PI3K/AKT signaling pathway activation. Because of crosstalk between Src and EGFR, it has been hypothesized that the suppression of Src and its downstream signaling partners may induce apoptosis [41] and overcome gefitinib resistance [38] in lung cancer cells. Therefore, the effects of NSC609077 on the expression of Src, STAT3, and ERK in both cell lines were further investigated. As shown in Figure 6B, NSC609077 exhibited weak or nonsignificant inhibitory effects on the total and phosphorylated expression of Src and STAT3 in H1975 cells and A549 cells exposed to EGF. Although the total ERK protein expression of these two cell lines was not inhibited by treatment with NSC609077, the level of phosphorylated ERK was significantly reduced in both cell lines, especially in A549 cells, exposed to EGF. Our results suggested that NSC609077 separately inhibits the expression or activation of the EGFR/PI3K/AKT axis, and ERK to suppress the growth and motility of H1975 cells and A549 cells.

## 3. Discussion

NSCLC is the most common malignant tumor worldwide, and it exhibits a high mortality rate [17]. Despite recent successes in the development of small-molecule inhibitors of EGFR kinase activity, this therapeutic strategy inevitably promotes the development of side effects [30] and drug-resistant mutants [27,31,32]. Therefore, the development of new therapeutic drugs is an urgent goal. In view of the mechanisms of action of various EGFR-TKIs, the construction of predictive models describing crucial pharmacophore characteristics of molecule candidates may improve the development and design of novel EGFR inhibitors.

In this study, we report a workflow of a multiple-stage virtual screening process that was used in conjunction with molecular docking to discover potential EGFR-targeted inhibitors for the suppression of EGFR activity. Initially, we developed quantitative (3D QSAR-based) and qualitative (common feature-based) pharmacophore models to identify pivotal chemical features of known EGFR inhibitors. Then, structure-based pharmacophore features were generated on the basis of ATP competitive sites in the EGFR L858R and T790M mutant structures. Then, a sequential virtual screening workflow used with three kinds of pharmacophores was performed to identify candidate compounds with the aforementioned crucial pharmacophore features.

For construction of the 3D QSAR pharmacophore model, 33 training set compounds were obtained from the BindingDB database, representing a wide range of IC_50_ values, across five orders of magnitude (0.1–5500 nM). The training set compounds that fit the following specific criteria were used to construct good hypothetical pharmacophores: (a) activity values ranged through at least four orders of magnitude; (b) the same targets were bound through a similar mechanism; and (c) activity values were measured with homogeneous procedures [42,43]. The ten best hypothetical models were constructed on the basis of the training set molecules. The top-ranked hypothetical model (Hypo1) was selected based on the following necessary rules: (i) the highest correlation coefficient, (ii) the lowest RMS value, (iii) the lowest total cost, (iv) a fixed cost similar to the total cost, (v) a high cost-difference, and (vi) a configuration cost value less than 17 [42,43]. Finally, one HBA, one HBD, and one HY chemical feature comprised the Hypo1 hypothetical model, as validated by Fischer’s randomization and test set methods.

During virtual screening, a CFP model was employed to account for key pharmacophore features of clinically approved EGFR-TKIs. The CFP model was generated on the basis of the common features among four clinical EGFR-TKIs: gefitinib, erlotinib, afatinib, and osimertinib. The four EGFR inhibitors exerted either reversible or irreversible inhibition to prevent the phosphorylation of EGFR-TKs, implying that the key chemical features significantly differ between the four inhibitors. For this reason, we adopted the CFP model as the structural query to perform virtual screening. This screening strategy is similar to that of previously published studies in which potential lead compounds that target EGFR were identified [44,45]. In addition, two SBP models derived on the basis of the ATP-bound pocket on the TK domain in the EGFR L858R and T790M mutants were also adopted in the screening processes to provide more comprehensive information on the crucial pharmacophore features. Because the ligand–receptor interaction depends on polar regions and hydrophobic regions, typical chemical features considered in designing a pharmacophore are a hydrogen bond acceptor, a hydrogen bond donor, a hydrophobic moiety, an aromatic ring moiety, and cation or anion moieties [46]. Therefore, a well-defined pharmacophore model was mainly comprised of hydrophobic volumes and hydrogen bond vectors. In this study, three types of chemical features, HBAs, HBDs, and HYs, were widely identified in the ligand- and structure-based pharmacophores and applied in the further virtual screening.

By combining the three types of pharmacophore models with molecular docking, we were able to perform a multiple-stage virtual screening on a dataset of NCI chemicals to identify potential EGFR inhibitors. As a result of the virtual screening, six novel EGFR inhibitors with desirably druggable properties, physicochemical properties, and good binding affinity for EGFR were identified; hence, these inhibitors were used in further in vitro experiments. To demonstrate the feasibility and validity of the multiple-stage PBVS, ELISAs were performed to validate the suppressive effect of the four available candidate compounds on EGFR activity in H1975 cells. Among these compounds, three compounds, NSC342715, NSC622394, and NSC609077, inhibited the phosphorylation of EGFR by 50% in the gefitinib-resistant H1975 lung cancer cell line (Table 4). Despite the fact that NSC7521 only inhibited close to 25% of EGFR activation in our results, a prior study has revealed that it can effectively suppress the EGFR phosphorylation in H1975 cells by using in vitro and in vivo experiments [47]. This result shows that the proposed PBVS workflow can be effectively used to identify the key pharmacophore characteristics of EGFR inhibitors. In addition, NSC609077 showed the highest inhibitory rate, suppressing EGFR phosphorylation by approximately 80%. NSC609077 inhibited EGFR phosphorylation in gefitinib-resistant H1975 cells and gefitinib-insensitive, but EGF-stimulated A549 cells (Figure 6A). Thus, NSC609077 was identified as a promising EGFR-targeted inhibitor candidate, and its functionality as an antitumor drug is considered worthy of investigation. To the best of our knowledge, no previous studies have reported the molecular mechanism and/or anticancer effect of NSC609077. Our results indicated that NSC609077 exerted a significant cytotoxic effect at the micromolar scale in H1975 cells and A549 cells. Moreover, the low dose of NSC609077, at a concentration of approximately 1 µM, also demonstrated a significant inhibitory effect on the colony formation and migration of these two types of lung cancer cells.

Previous studies have reported increased activation of the EGFR/PI3K/AKT signal transduction pathway in EGFR-TKI-resistant NSCLC cells, and this activation promotes cell proliferation and suppresses the apoptosis of cancer cells [39,40,48,49]. Hence, a few studies have attempted to overcome the resistance of gefitinib in NSCLC cells through the suppression of PI3K/AKT-related signaling by treatment with synergistic drugs [38,50] or a single drug [51]. Our results showed that NSC609077 can inhibit the phosphorylation of the EGFR/PI3K/AKT signaling pathway in H1975 cells, but it had no effect in A549 cells. In contrast, NSC609077 exerted a suppressive effect on the phosphorylation of ERK in both lung cancer cell lines, especially in A549 cells. A previous study found that the proliferation-suppressive effect was increased on A549 cells harboring wild-type EGFR when the MEK/ERK pathway was blocked [38]. Overall, we speculated that NSC609077 shows the potential to be an EGFR-targeted drug that may be used to overcome EGFR-TKI resistance in NSCLC by simultaneously blocking PI3K/AKT and MEK/ERK pathways, a concept that has also been previously proposed [52]. Importantly, further exploration into the mechanisms of NSC609077 action might contribute to the development of novel EGFR-targeted inhibitors for NSCLC treatment.

In conclusion, we constructed a rational workflow for multiple-stage PBVS and performed the ELISA experiments to identify important features of EGFR kinase inhibitors and to discover four potential EGFR-targeted inhibitors. Among the four compounds, NSC609077 showed a significant inhibitory effect on the EGFR activity and inhibited lung cancer cell viability, proliferation, and migration by suppressing EGFR-related signaling pathways. The results showed that the application of different pharmacophore models derived from various EGFR inhibitors or protein structures may be useful in facilitating the discovery of novel EGFR-targeted drugs in the future.

## 4. Materials and Methods

### 4.1. 3D QSAR Pharmacophore Generation and Evaluation

Training and test sets comprising 33 and 34 compounds, respectively, were collected and employed to construct the 3D QSAR pharmacophore model. Four types of chemical features (HBAs, HBDs, HYs, ARs) were identified from among the training set molecules to generate a hypothetical pharmacophore. The 3D QSAR pharmacophore generation protocol in DS3.5 (the HypoGen algorithm) was adopted to produce a series of hypothetical pharmacophores based on crucial chemical features and activity values of the training set molecules. HypoGen generates hypothetical models based on commonalities among the active molecules, excluding similarities with inactive molecules, and optimizes the solution by simulated annealing [42]. The 10 most relevant hypothetical structures and significant statistical parameters were generated. The top-ranked model was selected as the optimal model and then evaluated using two methods: test set validation for the evaluation of predictive power and Fischer’s randomization test for judging the significance of the model.

### 4.2. Common Feature Pharmacophore Modeling

The common feature pharmacophore generation protocol (the HipHop algorithm) [53] was employed to identify the vital chemical features of clinical EGFR-TKIs, such as gefitinib, erlotinib, afatinib, and osimertinib. The HipHop approach was used to detect common features in these four compounds for the generation of a CFP model comprising key features, including HBAs, HBDs, HYs, and ARs. The 10 highest-ranked hypothetical models based on the corresponding parameters were generated. The best model was chosen based on the higher score and most significant parameters.

### 4.3. Structure-Based Pharmacophore Modeling

Crystal structures of the EGFR-TK domain with the exon 21 L858R mutation (Protein Data Bank (PDB) ID: 2ITV) [54] and exon 20 T790M mutation (PDB ID: 2JIT) [55] were downloaded from the PDB database. Water molecules were removed from the protein structure, and hydrogen atoms and charge potential information were added. The ligand-bound pocket in the TK domain was predefined as an active pocket. The interaction generation protocol [56,57] was applied to characterize the chemical features in the active pocket of two individual protein structures. The density parameters of the polar sites and lipophilic sites were both set to 25 for identification of the hydrophilic and hydrophobic features, respectively. The estimated features were then clustered and optimized on the basis of their interaction patterns.

### 4.4. Pharmacophore-Based Virtual Screening (PBVS)

After construction and evaluation, three pharmacophore models were applied to perform multiple-stage PBVS with the NCI chemical database. The compounds were prepared by using the prepare ligand protocol. The conformations of each compound were generated using the best conformer generation method. The ligand pharmacophore mapping protocol in DS3.5 was employed with the rigid-body fitting method to screen for 3D QSAR pharmacophores and CFPs. In addition, the screening library protocol was utilized for pharmacophore mapping of the SBP to retained hits that had been properly mapped to ligand-based pharmacophores. The fit values were calculated on the basis of different types of pharmacophore models and adopted as the criteria to select hit compounds. A high fit value indicated a greater overlap between the compound and the pharmacophore features and vice versa.

### 4.5. Molecular Docking Studies

A molecular docking analysis was performed to refine the list of hit compounds obtained from multiple-stage PBVS. The LibDock docking algorithm [58] in DS3.5 was applied to simulate the docking poses of each compound. The conformations of each compound were aligned to polar and nonpolar interaction sites on the predefined active pocket within the EGFR-TK domain structure; the docking scores were then calculated. The best docking conformation for each compound, which was determined based on the highest docking score value and the best orientation in the binding pocket, was saved. The docking score for each compound was adopted as the criterion to include a candidate in further in vitro biological assays.

### 4.6. Cell Culture and Drug Treatment

The H1975 (ATCC CRL-5908) and A549 (ATCC CCL-185) human lung cancer cell lines were purchased from the American Type Culture Collection (ATCC, Manassas, VA, USA). H1975 cells harbor both the L858R in exon 21 and T790M in exon 20 mutations in the EGFR gene, and A549 cells carry wild-type EGFR. The cells were maintained at 37 °C in a humidified atmosphere with 5% CO_2_ and cultured in RPMI-1640 medium (Gibco, Carlsbad, CA, USA) supplemented with 10% fetal bovine serum (FBS; Gibco, Carlsbad, CA, USA) and 1% penicillin/streptomycin (Gibco, Carlsbad, CA, USA). The candidate compounds were acquired from the NCI (Bethesda, MD, USA) and prepared in dimethyl sulfoxide (DMSO) stock solution. The vehicle control was treated with 0.1% DMSO.

### 4.7. Enzyme-Linked Immunosorbent Assay (ELISA)

DuoSet Human Phospho-EGFR (Y1068) ELISA reagent (R&D Systems, Minneapolis, MN, USA) was used according to the manufacturer’s instructions to detect the EGFR phosphorylation level. Lysate of H1975 cells treated with compound or vehicle for 24 h was added to a 96-well plate precoated with a capture antibody to bind phosphorylated EGFR (phospho-EGFR) and unphosphorylated EGFR. Then, the levels of phospho-EGFR^Y1068^ were assessed using an immobilized antibody reacted with a horseradish peroxidase (HRP)-conjugated antibody. The antibody reaction was developed by adding NeA-Blue tetramethylbenzidine substrate (Clinical Science Products, Bristol County, MA, USA) and stopped with 2 N H_2_SO_4_. The absorbance was measured at 450 nm against a reference wavelength of 570 nm with a Victor3 spectrophotometer (PerkinElmer, Santa Clara, CA, USA).

### 4.8. Cell Viability Assay

PrestoBlue cell viability reagent (Invitrogen, Carlsbad, CA, USA) was used to assess the cytotoxic effect of candidate compounds. A549 and H1975 human lung cancer cells were treated with the compounds at different concentrations for 48 h. Then, PrestoBlue reagent was added to the cells, and the absorbance at 570/600 nm was measured with a Victor3 spectrophotometer (PerkinElmer, Santa Clara, CA, USA). The 10, 20, and half-maximum inhibitory concentrations, that is, the IC_10_, IC_20_, and IC_50_ values, respectively, were calculated with CalcuSyn software (Version 2.1; Biosoft, Cambridge, UK).

### 4.9. Clonogenicity Assay

An anchorage-dependent growth assay was performed to observe the suppression efficacy of candidate compounds on lung cancer cell colony formation. A total of 500 cells were seeded in each six-well plate containing culture medium and a drug solution for 7–10 days. Then, the cells were washed with 1× PBS and fixed with 4% paraformaldehyde. The cells were subsequently stained with 0.05% crystal violet. A picture of each well was taken with an inverted microscope. The mean value of each colony-covered area was determined with ImageJ software (National Institutes of Health, Bethesda, MD, USA). Each treatment effect was determined by the percentage of the covered area.

### 4.10. Scratch-Wound Assay

A wound-healing assay was performed to assess the effects of candidate compounds on lung cancer cell migration, as previously described [59]. Cells were seeded into six-well plates containing culture medium and drug solution for 48 h. Subsequently, the attached cell monolayer was scratched with a 20 μL pipette tip. The images of the wound healing were captured with an inverted microscope every 4 h for 16 h and analyzed with ImageJ software. The percentage of the cell coverage area was measured and compared with the value calculated at 0 h. An increase in the percentage of cell coverage area indicated the degree of migrated cells.

### 4.11. Western Blot Analysis

Western blotting was performed to determine the expression of EGFR signaling pathway-related proteins in lung cancer cells treated with candidate compounds, as described previously [60]. H1975 cells were seeded in culture medium and drug solution for 48 h. A549 cells were pretreated with candidate compounds at desired concentrations for 48 h before exposure or non-exposure to 20 ng/mL EGF for 30 min. The cells were then lysed in buffer containing 50 mM Tris-HCl (pH 7.4), 150 mM NaCl, 1 mM EDTA, and 1% NP-40. The cell lysates were subjected to Western blotting. Recombinant human EGF was purchased from Cascade Biologics (Portland, OR, USA). Antibodies against phospho-EGFR (Tyr1068), phospho-PI3K (Tyr199/Tyr458), phospho-STAT3 (Tyr705), phospho-STAT3 (Ser727), and AKT were purchased from Cell Signaling Technology (Beverly, MA, USA). Antibodies against EGFR, PI3K, STAT3, ERK, GAPDH, and phospho-ERK (Tyr204) were purchased from Santa Cruz Biotechnology (Dallas, TX, USA). Antibodies against phospho-AKT (Ser473) were purchased from Millipore (Billerica, MA, USA). Antibodies against Src were produced in our laboratory (ATCC CRL-2651). Antibodies against phospho-Src (Tyr418) were purchased from Invitrogen (Carlsbad, CA, USA). GAPDH was used as the loading control. Protein expression was quantified with ImageJ software.

### 4.12. Statistical Analysis

All experiments were performed in at least duplicate or triplicate, and statistical analysis was carried out on the results via a Student’s *t*-test or ANOVA (Excel 2016; Microsoft, Redmond, Washington, USA). A *p* value < 0.05 indicates statistical significance compared with the control.

## Figures and Tables

**Table 1:** The ten best 3D QSAR hypothetical pharmacophores constructed using a training set against EGFR inhibitors.

Pharmacophore Hypothesis	Total Cost	Cost Difference	RMS	Correlation	Features
Hypo1	136.11	60.98	0.88	0.92	HBA, HBD, HY
Hypo2	136.27	60.82	0.90	0.91	HBA, HBD, HY
Hypo3	144.36	52.73	1.14	0.87	HBA, HBD, HY
Hypo4	146.30	50.79	1.19	0.85	HBA, HBD, HY
Hypo5	147.97	49.12	1.21	0.85	HBD, HY, RA
Hypo6	150.20	46.89	1.28	0.83	HBA, HBD, HY
Hypo7	150.36	46.73	1.25	0.84	HBD, HY, RA
Hypo8	150.65	46.44	1.30	0.82	HBA, HBD, HY
Hypo9	150.97	46.12	1.30	0.82	HBD, HY, RA
Hypo10	151.64	45.45	1.27	0.84	HBD, HY, RA

Cost difference: The difference between the null and total costs. The null cost, the fixed cost, and the configuration cost are 197.09, 122.56, and 10.43, respectively. All costs are reported as bit units. RMS: root mean square deviation. Features—HBA, hydrogen bond acceptor; HBD, hydrogen bond donor; HY, hydrophobic region; RA, ring aromatic.

**Table 2:** The experimental and predicted activities of 33 training set compounds calculated on the basis of the 3D QSAR-derived Hypo1 model.

BindingDB Code	Experimental IC_50_ nM	Predicted IC_50_ nM	Error	Experimental Scale ^‡^	Predicted Scale ^‡^	3D-QSAR Fit Value
BDBM3297	0.1	0.4	4.00	+++	+++	6.79
BDBM3520	0.3	0.4	1.33	+++	+++	6.77
BDBM3522	0.4	0.4	1.00	+++	+++	6.80
BDBM3583	0.4	0.7	1.75	+++	+++	6.58
BDBM3294	0.8	2.2	2.75	+++	+++	6.07
BDBM3519	2.0	9.3	4.65	+++	+++	5.45
BDBM3780	2.7	3.5	1.30	+++	+++	5.87
BDBM3546	3.8	2.4	−1.58	+++	+++	6.04
BDBM3535	4.0	2.0	−2.00	+++	+++	6.12
BDBM3538	4.7	0.4	−11.75	+++	+++	6.79
BDBM3767	9.4	20.4	2.17	+++	+++	5.11
BDBM3781	20.0	66.0	3.30	+++	+++	4.60
BDBM3263	23.0	75.1	3.27	+++	+++	4.54
BDBM3264	27.0	72.8	2.70	+++	+++	4.55
BDBM3753	31.0	55.5	1.79	+++	+++	4.67
BDBM3508	55.0	73.2	1.33	+++	+++	4.55
BDBM3503	56.0	164.8	2.94	+++	++	4.20
BDBM3283	58.0	81.6	1.41	+++	+++	4.50
BDBM3763	58.0	209.8	3.62	+++	++	4.09
BDBM3744	72.0	63.6	−1.13	+++	+++	4.61
BDBM3265	80.0	72.0	−1.11	+++	+++	4.56
BDBM3536	84.0	70.5	−1.19	+++	+++	4.57
BDBM3518	100.0	16.8	−5.95	++	+++	5.19
BDBM3516	120.0	79.8	−1.50	++	+++	4.51
BDBM3745	132.0	85.6	−1.54	++	+++	4.48
BDBM3292	139.0	90.7	−1.53	++	+++	4.46
BDBM3757	147.0	57.4	−2.56	++	+++	4.66
BDBM3752	264.0	188.8	−1.40	++	++	4.14
BDBM3256	320.0	106.8	−3.00	++	++	4.39
BDBM3259	344.0	202.1	−1.70	++	++	4.11
BDBM3295	348.0	69.2	−5.03	++	+++	4.58
BDBM3510	770.0	228.5	−3.37	++	++	4.06
BDBM3269	5500.0	3769.7	−1.46	+	+	2.84

Error: The difference between true and estimated activity. A predicted half maximum inhibitory concentration IC_50_ that is higher than the experimental IC_50_ is indicated by the “+” sign; in contrast, a predicted IC_50_ that is lower than the experimental IC_50_ is indicated by the “−” sign. A value of 1 indicates that the predicted IC_50_ is equal to the experimental IC_50_. ^‡^ Activity scale: +++, highly active (IC_50_ < 100 nM); ++, moderately active (100 nM ≤ IC_50_ < 1000 nM); +, nominally active or inactive (IC_50_ ≥ 1000 nM). 3D-QSAR fit value: The fit value indicates the extent to which the features of the 3D QSAR Hypo1 model overlap the chemical features of the training set molecule.

**Table 3:** The ten best common feature-derived hypothetical pharmacophores constructed on the basis of four clinically approved EGFR inhibitors.

Pharmacophore Hypothesis	Rank	Direct Hit	Partial Hit	Max Hit	Features
Pharm1	54.25	1101	0010	6	RRHHAA
Pharm2	53.04	1111	0000	6	HHHDAA
Pharm3	52.64	1101	0010	6	RRHHAA
Pharm4	52.48	1111	0000	5	RRHAA
Pharm5	52.32	1101	0010	6	RRHHAA
Pharm6	52.32	1101	0010	6	RRHHAA
Pharm7	52.25	1101	0010	6	RRHHAA
Pharm8	52.25	1101	0010	6	RRHHAA
Pharm9	52.20	1111	0000	5	RRHAA
Pharm10	52.20	1111	0000	5	RRHAA

Features: A, hydrogen bond acceptor; D, hydrogen bond donor; H, hydrophobic area; and R, ring aromatic.

**Table 4:** The pharmacophore-derived fit values, docking scores, and the suppression effects of the candidate compounds on EGFR phosphorylation at Y1068.

NSC Number	3D-QSAR Fit Value	CFP Fit Value	SBP Fit Value		LibDock Score		Phosphorylation Level (%)	Inhibitory Rate (%)
			EGFR p.L858R (PDB: 2ITV)	EGFR p.T790M (PDB: 2JIT)	EGFR p.L858R (PDB: 2ITV)	EGFR p.T790M (PDB: 2JIT)		
NSC7521	6.00	3.18	2.89	2.57	124.05	122.70	75 ± 15	25 ± 15
NSC342715	6.47	3.26	2.98	2.65	123.30	119.98	37 ± 6 *	63 ± 6 *
NSC622394	6.05	3.37	1.97	2.54	148.73	123.90	32 ± 8 *	68 ± 8 *
NSC609077	6.22	2.79	2.77	1.68	145.57	152.88	21 ± 6 *	79 ± 6 *
NSC622442	6.68	3.73	3.12	2.49	129.85	124.22	NA	NA
NSC623897	6.03	3.77	2.48	3.33	117.06	116.17	NA	NA

* *p* < 0.05 indicates statistical significance compared with the vehicle control. NA, not available (the corresponding compound was not in the NCI open chemicals repository). NSC Number: National service center number. Abbreviations- 3D-QSAR: 3D quantitative structure-activity relationship, CFP: Common feature pharmacophore, SBP: Structure-based pharmacophore, *EGFR*: Epidermal growth factor receptor, PDB: Protein data bank.

## Data Availability

Data is contained within the article and appendices.

## Appendix A

**Table A1:** The pharmacophore-derived fit values and docking scores of clinically approved EGFR inhibitors.

EGFR-TKI	3D-QSAR Fit Value	CFP Fit Value	SBP Fit Value		LibDock Score	
			EGFR p.L858R (PDB: 2ITV)	EGFR p.T790M (PDB: 2JIT)	EGFR p.L858R (PDB: 2ITV)	EGFR p.T790M (PDB: 2JIT)
Erlotinib	**3.17**	**1.89**	**1.50**	**1.15**	116.60	110.71
Gefitinib	3.92	3.28	2.49	2.29	113.73	**106.28**
Afatinib	5.89	2.90	2.32	2.49	**111.59**	107.47
Osimertinib	4.86	6.00	2.99	3.34	115.66	115.27

Note: Bold indicates values used as criteria during the multiple-stage pharmacophore-based virtual screening coupled with molecular docking. Abbreviations- *EGFR*: Epidermal growth factor receptor, EGFR-TKI: Epidermal growth factor receptor tyrosine kinase inhibitor, 3D-QSAR: 3D quantitative structure-activity relationship, CFP: Common feature pharmacophore, SBP: Structure-based pharmacophore, PDB: Protein data bank.
